# Phenolic Compounds Protect Cultured Hippocampal Neurons against Ethanol-Withdrawal Induced Oxidative Stress

**DOI:** 10.3390/ijms10041773

**Published:** 2009-04-20

**Authors:** Katalin Prokai-Tatrai, Laszlo Prokai, James W. Simpkins, Marianna E. Jung

**Affiliations:** 1 Department of Pharmacology & Neuroscience, University of North Texas Health Science Center, Fort Worth, TX, USA; E-Mails: jsimpkin@hsc.unt.edu (J.W.S.); mjung@hsc.unt.edu (M.E.J.); 2 Department of Molecular Biology & Immunology, University of North Texas Health Science Center, Fort Worth, TX, USA; E-Mail: lprokai@hsc.unt.edu

**Keywords:** Ethanol withdrawal, Lipid peroxidation, Oxidative stress, Phenolic antioxidant, Protein carbonylation

## Abstract

Ethanol withdrawal is linked to elevated oxidative damage to neurons. Here we report our findings on the contribution of phenolic antioxidants (17β-estradiol, *p*-octyl-phenol and 2,6-di-*tert*-butyl-4-methylphenol) to counterbalance sudden ethanol withdrawal-initiated oxidative events in hippocampus-derived cultured HT-22 cells. We showed that ethanol withdrawal for 4 h after 24-h ethanol treatment provoked greater levels of oxidative damage than the preceding ethanol exposure. Phenolic antioxidant treatment either during ethanol exposure or ethanol withdrawal only, however, dose-dependently reversed cellular oxidative damage, as demonstrated by the significantly enhanced cell viability, reduced malondialdehyde production and protein carbonylation, compared to untreated cells. Interestingly, the antioxidant treatment schedule had no significant impact on the observed neuroprotection. In addition, the efficacy of the three phenolic compounds was practically equipotent in protecting HT-22 cells in spite of predictions based on an *in silico* study and a cell free assay of lipid peroxidation. This finding implies that free-radical scavenging may not be the sole factor responsible for the observed neuroprotection and warrants further studies to establish, whether the HT-22 line is indeed a suitable model for *in vitro* screening of antioxidants against EW-related neuronal damage.

## 1. Introduction

Neurotoxicity induced by ethanol causes tissue damage to a variety of organs in humans, a finding which has been corroborated by numerous studies using animal models [1]. A major cause of this detrimental effect is believed to be elevated oxidative stress levels *via* ethanol-induced generation of reactive oxygen species and impairment of the antioxidant defense system [2].

The brain is especially sensitive to oxidative stress, because it has high oxidative metabolic rate and low levels of antioxidant enzymes compared to other tissues [3]. Additionally, it contains high concentrations of redox-active metals [4] that may promote Fenton and Fenton-like reactions [5]. Large amount of unsaturated lipids are also present in the brain that are easy targets for free radicals because they provide readily removable hydrogens [6]. The resulting lipid radicals then rapidly react with molecular oxygen to form peroxyl radicals. This lipid peroxidation (LPO) initiates a series of complex, autocatalytic propagation reactions that generate a variety of thiobarbituric acid reactive substances (TBARs) such as the genotoxic malondialdehyde (MDA), as their end products [7]. Like free radicals, MDA and related carbonyl compounds are highly reactive and could enhance or extend the cellular damage caused by localized radical reactions. On the protein level, they give rise to protein carbonyls [8,9], further disrupting normal functions of the organism.

Unfortunately, not only chronic ethanol exposure, but also abrupt withdrawal can produce significant neurotoxicity due to oxidative stress [10–12]. While ethanol itself is pro-oxidant because it directly generates reactive oxygen species during its metabolism [13], ethanol withdrawal (EW) produces oxidative stress indirectly through the activation of excitatory neurotransmitter receptors and the concomitant alteration of intracellular calcium levels [14]. It has been shown that sudden EW results in an increase in glutamate levels/glutamate receptor activity and a compensatory up-regulation of *N*-methyl-D-aspartate (NMDA) receptors [15,16] as well as in a decrease in GABA levels/GABA-receptor activity [17,18]. These events will eventually lead to oxidative stress [19]. Therefore, free-radical scavenging antioxidants may be beneficial to alleviate oxidative stress-initiated neuronal cell death during EW.

**Figure 1.**
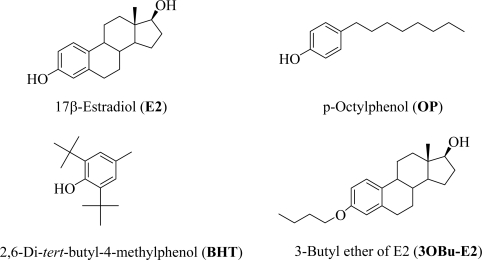
Chemical structures of E2, OP, BHT and 3OBu-E2.

A plethora of evidence support that estrogens are powerful neuroprotectants. It has been established that the involvement of the nuclear estrogen receptors are not necessary to achieve neuroprotection [20]. Estrogens enhance cell survival by suppressing the neurotoxic stimuli partly *via* their direct radical-scavenging antioxidant activity [5]. We have previously shown that 17β-estradiol (E2, Figure 1) protects against insults associated with EW at neuronal, behavioral and signal transduction levels in rats [21–23]. Brain tissues obtained from female rats showed increased LPO after EW in a manner that correlated with behavioral impairment, but was prevented with treatment by E2. We have also recently adopted an *in vitro* paradigm, the HT-22 immortalized hippocampal cell line, as a cellular model for EW [24,25]. We found that E2 treatment provided protection against cell death and LPO induced by EW toxicity. In the present exploratory study, we extended our investigation on the contribution of non-steroidal phenolic antioxidants to reduce the adverse effects of EW on this cell line. Specifically, we compared the efficacy of E2 with that of *p*-octylphenol (OP) and 2,6-di-*tert*-butyl-4-methylphenol (BHT), known phenolic antioxidants, to enhance HT-22 cell viability and reduce cellular oxidative damage upon EW. Here the antioxidant treatments started either simultaneously with the ethanol exposure [24] or only with EW. Control experiments were also done using an E2-derivative lacking the free phenolic OH believed to be the quintessential feature to achieve free radical scavenging antioxidant activity by phenolic compounds [26,27]. Additionally, an *in silico* study and a well-established cell-free assay of LPO [28,29] have also been done to predict the antioxidant potency of the test compounds.

## 2. Results and Discussion

### 2.1. Computational and cell-free inhibition of LPO studies

The chemical structures of phenolic compounds used in the present studies are shown in Figure 1. Earlier, we have found that the protective effect of estrogens against EW-induced neurotoxicity is in part due to their antioxidant activity [5], classifying them as simple phenolic antioxidants in this regard [20,27]. The antioxidant property of such compounds is attributed to the presence of the free phenolic OH group. Therefore, first an *in silico* prediction of the antioxidant potency of the test compounds was done by calculating the bond dissociation enthalpy (BDE) of the phenolic O-H bond (Table 1). BDE of this bond is one of the most important predictors for the antioxidant potency [30–32] because this type of antioxidants exerts their action *via* an initial hydrogen transfer reaction to free radicals. The rate constant for this process depends on the strength of the O-H bond. As such, the potency of a phenolic antioxidant usually correlates well with BDE; although other factors, such as lipophilicity may also influence the free radical scavenging potency. (The test compounds used in our studies are all highly lipophilic molecules; logP of E2 is around 4 [33] and is approximately 1 log unit higher for BHT and OP [34,35]). For the polycyclic estrogens, planarity has also been proposed as a contributing factor contributing to cytoprotection [27].

Table 1 shows that based on the calculated BDE values BHT is projected to be the most potent free radical scavenging antioxidant (BDE = 77.0 kcal/mol), while E2 and OP may be considered equipotent. As mentioned above, LPO is a common marker of oxidative damage; therefore, we employed a widely used cell-free assay [28,29] to predict the capacity of E2, OP and BHT to inhibit LPO using linoleic acid as a lipid model. We chose this cell-free model because we first wanted to experimentally assess the “pure” antioxidant potency elicited by our selected test compounds. With both the ferric thiocyanate (FTC, [36]) and TBARS [37] methods, the inhibition of LPO is measured in the presence of various concentrations of the test compounds. The FTC method measures the amount of peroxides in initial stages of autooxidation. During the oxidation process, peroxides gradually decompose to carbonyls such as MDA which are measured with the TBARS method. Therefore, the two assays are complementary when they are used to evaluate antioxidant capacity to inhibit LPO.

In the cell free model, BHT showed the highest potency for suppressing the autooxidation of linoleic acid with both the FTC and TBARS methods, although with the latter method a similar IC_50_ was measured for E2 (Table 1). The high potency of BHT is not unexpected considering it is a hindered orto-disubstituted phenol derivative known to be a more effective antioxidant compared to the monosubstituted counterpart [38]. Altogether, the cell-free model for LPO has confirmed the *in silico* prediction regarding antioxidant potency of the test compounds based on BDE values.

### 2.2. Oxidative stress by continuous ethanol exposure and its subsequent removal on HT-22 cells

**Figure 2.**
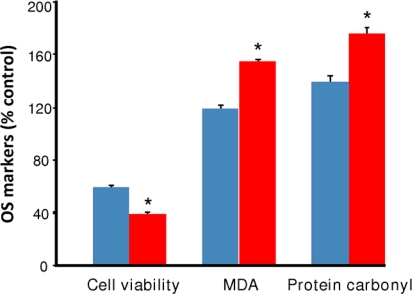
Oxidative stress (OS) markers on HT-22 cells after 24-h ethanol (EtOH, 100 mM) exposure and subsequent 4-h ethanol withdrawal (EW). In control experiments (vehicle only) cell viability was counted as 100%, MDA level was 173 ± 6.5 nmol/mg protein and protein carbonyl content was 1.28 ± 0.01 nmol/mg protein. Data are given as mean ± S.E.M. for n=4. Both ethanol exposure ( 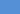) and EW ( 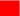), produced a statistically significant difference (p<0.001) in OS markers from those measured in control experiments. Asterisk indicates a significant difference (p<0.01) between EW *vs* EtOH exposure only.

When the HT-22 cellular model was exposed to a steady concentration of 100 mM ethanol for 24 h as reported earlier [11,24,25,39], the cell viability (expressed as % of control and quantified by the Calcein AM assay [24–26]) decreased significantly compared to the ethanol-free control cells (counted as 100%), as shown in Figure 2. Simultaneously, the levels of MDA and protein carbonylation enhanced significantly compared to those of the control cells. The cellular oxidative damage was even more profound when alcohol was suddenly removed and replaced with the culture media (Figure 2). EW further amplified LPO and protein carbonylation compared to those of the ethanol-treated cells, showing that EW, indeed, causes more oxidative damage in our *in vitro* model than a steady concentration of prolonged ethanol exposure. A similar tendency was also observed at higher concentration of alcohol exposure, while further extending the time of alcohol treatment did not significantly changed the outcome of oxidative markers (data not shown). As such, our data confirmed previous findings [11,24,25] that sudden EW eventually produces more oxidative stress than continuous ethanol treatment *per se*; therefore, this cell line is a more suitable model for EW than ethanol exposure.

### 2.3. Effect of phenolic antioxidants on cell survival of EW-subjected HT-22 cells

**Figure 3.**
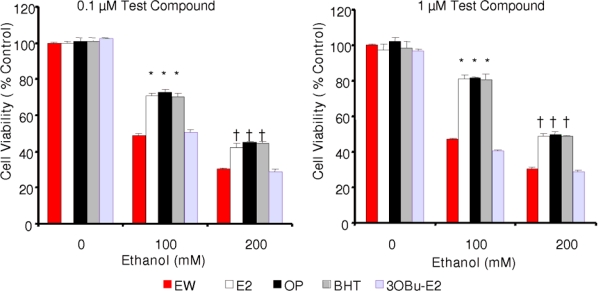
Effect of E2, OP, BHT and 3OBu-E2 on EW-induced cytotoxicity. Data are given as mean ± S.E.M. (n=4),*p<0.001 and †p<0.01 vs. corresponding EW control (no test compound) experiments.

In one set of experiments, HT-22 cells were treated with one of the phenolic antioxidants (E2, BHT or OP) at 0.1 μM or 1 μM concentration (in dimethyl sulfoxide, DMSO) at the same time the cells were exposed to the 24-h neurotoxic insult (100 mM or 200 mM ethanol, respectively). Following the 4-h EW [24], cell viability was assessed. Figure 3 shows, that there was a significant and dose-dependent increase in cell survival upon treating the cells with E2, OP or BHT at both doses of ethanol compared to control cells (EW cells without antioxidants treatment). At 0.1 μM antioxidant concentration cell viability increased by approximately 40% and at 1 μM dose by approximately 50%, respectively after both concentrations of ethanol treatment compared to the control. Surprisingly, these phenolic compounds were essentially equipotent to rescue the cells against the toxic insult, in spite of predictions shown in Table 1.

In order to demonstrate the crucial role of the free phenolic OH in reducing EW-induced oxidative stress by free radical scavenging antioxidant action, we also tested an E2 derivative, 3OBu-E2 (Figure 1) in which the phenolic OH is blocked by forming its butyl ether [26]. As expected, 3OBu-E2 was ineffective at counterbalancing the damaging effect of EW on cell survival, due to this compound’s inability to function as a phenolic antioxidant. In another set of experiments, antioxidant treatments started concomitantly with EW only; i.e., after the alcohol was removed and replaced with vehicle-containing medium and test compound. Notably, we obtained no significant difference in cell survivals (data not shown) from those measured after the co-treatment schedule described above (Figure 3). As such, E2, OP and BHT were basically equipotent at both concentrations (0.1 μM and 1.0 μM) demonstrating significant (p<0.001) and dose-dependent cell survival enhancement at both concentrations of ethanol exposure (100 mM and 200 mM, respectively), independently from the antioxidant treatment schedule. This observation may suggest that cells can be treated concomitantly with EW only to ameliorate cellular damage.

### 2.4. Effect of phenolic antioxidants on EW-induced lipid peroxidation

**Figure 4.**
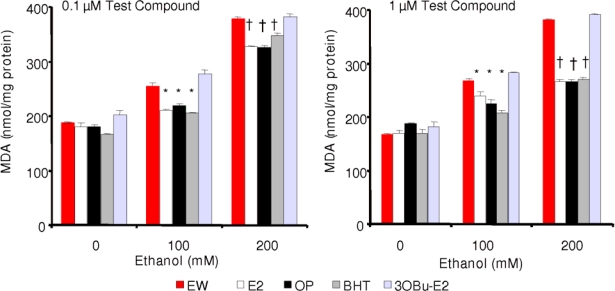
Effect of E2, OP, BHT and 3OBu-E2 on EW-induced lipid peroxidation. Data are given as mean ± S.E.M. (n=4). *p<0.001 and ^†^p<0.01 vs. corresponding EW control.

For exploring how EW induces LPO, cells were treated analogously to those of the cell survival studies. Without antioxidant treatment as shown in Figure 2, EW induced approximately a 1.5-fold increase in MDA production after 100 mM ethanol (and approximately a 2-fold increase after 200 mM ethanol exposure, data not shown) compared to that of the ethanol-free control group. When the cells were treated simultaneously with alcohol and a test compound E2, OP, or BHT, but not 3OBu-E2, were essentially equipotent in reducing LPO (Figure 4). For example, MDA levels decreased by approximately 15% and 30% at 200 mM ethanol exposure, when 0.1 μM and 1 μM of phenolic antioxidants were used, respectively. Moreover, in agreement with the cell survival studies, there was no significant effect of the treatments schedules; thus, antioxidant treatment during EW only produced practically the same decrease in LPO (data not shown) than co-treatment with EtOH (Figure 4). Additionally, there was no significant difference in the LPO–reducing potency of the phenolic test compounds under the experimental conditions used in our studies. This observation warrants further studies to ascertain the utility of HT-22 cells for testing various antioxidants to reduce LPO upon EW.

### 2.5. Effect of phenolic antioxidants on protein carbonylation

**Figure 5.**
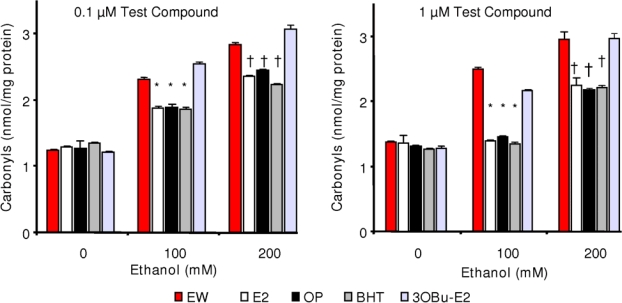
Effects of E2, OP, BHT and 3OBu-E2 on EW-induced protein carbonylation. Data are given as mean ± S.E.M. for n=4. *p<0.001 and ^†^p<0.01 vs. corresponding EW.

Consistent with the cell survival and LPO studies, antioxidant treatment had a profound effect on lowering protein carbonylation compared to the control cells (EW only), as shown in Figure. 5. Protein carbonylation in cells treated with E2, OP or BHT were significantly (p<0.01) lower than that of control cells (no antioxidant treatment after EW, Figure 2) or cells treated with 3-OBu-E2 at both doses of ethanol. As such, when e.g., 1.0 μM of antioxidant (E2, OP or BHT) treatment was used concomitantly with 100-mM ethanol exposure, the protein carbonyl levels decreased to approximately 1.4 nmol/mg protein from approximately 2.4 nmol/mg protein measured in the control cells. As with the cell survival and LPO studies, no statistically significant difference was observed in the protein carbonyl content among the antioxidants used here as well as between the treatment schedules (antioxidant treatment during ethanol exposure or during EW only) suggesting that antioxidant treatment is sufficient only during EW to prevent EW-induced oxidative damage to the cells.

## 3. Experimental Section

### 3.1. Materials

17β-Estradiol (E2) was purchased from Steraloids (New Port, RI). *p*-Octylphenol (OP) and 2,6-di-*tert*-butyl-4-methylphenol (BHT), as well as other chemicals used for cell culture studies were purchased from Sigma (St. Louis, MO). 3-*O*-Butyl-17β-estradiol (3OBu-E2) was prepared as reported before [26]. HT22 cells, a murine hippocampal cell line, were the generous gift of Dr. David Schubert (Salk Institute, San Diego, CA).

### 3.2. Computational studies

Bond dissociation enthalpies (BDEs) were calculated by a method incorporated into the window-based molecular modeling software BioMedCAChe (version 6.1, Fujitsu, Beaverton, OR) using PM5 semi-empirical quantum chemical approximation. Calculations were done after performing full geometry optimization (RMS gradient <0.1 kcal/[Å · mol]) and energy minimization by using the PM3 semi-empirical method with open-shell wave functions [unrestricted Hartree-Fock (UHF) approach], implemented in the BioMedCAChe (Fujitsu, Beaverton, OR) molecular modeling program (version 6.1).

### 3.3. Inhibition of lipid peroxidation

The capacity of the test compounds to inhibit LPO in a cell-free medium was studied according to published methods [28,29,40]. Incubation mixtures consisted of 2 mL of test compounds (at varying concentrations), 2.05 mL of 2.5% (v/v) linoleic acid in ethanol, 4 mL of 0.05 M phosphate buffer (pH 7.0) and 1.95 mL water, in a vial with a screw cap. Control experiments were also done using 2.05 ml EtOH only. The incubations were carried out in the dark at 40°C for nine days. Then, the samples were analyzed using both the FTC [36] and the TBARS methods [37]. For FTC, 0.1 mL incubation mixture and, then 0.1 mL of 30% (w/v) aqueous NH_4_SCN were added into 9.7 mL of 75% (v/v) aqueous ethanol. Thirty seconds after the addition of the NH_4_SCN, 0.1 mL of 0.02 M FeCl_2_ in 3.5% (v/v) HCl was added and 3 min later absorbance of the solution was measured. For TBARS, 0.4 mL 20% (w/v) trichloroacetic acid and 0.4 mL 0.67% (w/v) aqueous thiobarbituric acid were added to 0.2 mL of incubation mixture. After being kept at 90°C for 10 minutes, the sample was allowed to cool and a supernatant was obtained by centrifugation at 10,000 rpm for 40 minutes. The absorbance of the supernatant was measured at 532 nm. The percentage of inhibition was calculated as (1 – A/A_0_) * 100; where A was the absorbance of the solution containing the inhibitor and A_0_ represented the absorbance of the control. IC_50_ values were determined by non-lineal fitting to sigmoid dose–response profiles with the Scientist for Windows program (version 2.0, MicroMath, Salt Lake City, UT).

### 3.4. Cell culture

The HT-22 line was originally selected from HT4 cells that were immortalized from primary hippocampal neurons using a temperature-sensitive Small Virus-40 T antigen [41]. The cells were grown in Dulbecco’s Modified Eagle Medium, supplemented with 10% charcoal-stripped Fetal Bovine Serum (HyClone, Logan, UT) and gentamicin (50 μg/mL), at 37°C in an atmosphere containing 5% CO_2_ and 95% air. Protein contents were determined by the Bradford method [42] using BSA as a reference.

### 3.5. HT-22 cell treatments [24,25]

For the assessment of cell viability, HT-22 cells were plated into 96-well tissue culture plates at 4,000 cells per well in 100 μL of cell culture medium. The following day, the cells were continuously exposed to 0, 100 or 200 mM of alcohol for 24 hours. The culture plates or Petri dishes were tightly sealed with parafilm immediately after ethanol treatment to prevent its evaporation. For the continuous EtOH exposure condition, cell viability and oxidative markers were measured at the end of the 24-h ethanol exposure. In EW experiments, the ethanol-containing medium was replaced by vehicle-containing medium for 4 h immediately after the 24-h EtOH exposure [43,44]. No measurable alcohol could be detected under this condition of EW and the concentration of ethanol was steady during alcohol treatment. Additionally, no significant difference was measured in cell viability between the 24-h and 28-h ethanol treatment groups. At the end of the 4-h EW, cell viability and oxidative markers were measured. Stock solutions of test compounds were prepared at a concentration of 0.1 μM and 1.0 μM in DMSO and were given to cells either together with the 24-h EtOH exposure [24] or only at the beginning of EW. Vehicle-treated control cultures were exposed in parallel to the same concentration of DMSO present in the experimental cultures. There were no measurable effects of DMSO treatment on cell viability under these conditions. Additionally, in experiments using the phenolic compounds only (no alcohol exposure), no significant change in cell viability was measured compared to the vehicle-treated cells; therefore, these compounds are deemed to be non-toxic to the HT-22 cell line.

### 3.6. Calcein-AM viability assay

Cell viability was quantitated using the membrane-permeant Calcein-AM dye (Molecular Probes, Eugene, OR). Following the removal of the medium from the 96-well plates, the cells were rinsed once with phosphate-buffered saline (PBS, pH 7.4) and incubated in a solution of 2.5 μM Calcein-AM in PBS. Twenty minutes later, fluorescence was determined using a Bio-Tek FL600 microplate reader (Winooski, VT) with an excitation/emission filter set at 485/530 nM. Cell culture wells treated with methanol served as blanks.

### 3.7. MDA assay

For the assessment of MDA content, the cells were plated into Petri dishes (Greiner Bio-One, Monroe, NC) at approximately 106 cells per dish. The levels of MDA were assessed using a colorimetric assay (Calbiochem, San Diego, CA) in which a chromogenic reagent (1-methyl-2-phenylindole) reacts with MDA under acidic (HCl) condition and yields a stable chromophore with maximal absorbance at 586 nM. Cells treated with EtOH were washed with PBS, centrifuged (100,000 x g for 60 minutes at 4°C), and suspended in 1 mL of PBS. Cell lysis was then done by sonication. Cell homogenates and the chromogenic reagent were incubated at 45°C for 60 min then the samples were cooled off, and the absorbance was read at 586 nm.

### 3.8. Measurement of protein carbonyl formation

For the assessment of carbonyl content the cells were plated into Petri dishes (Greiner Bio-One, Monroe, NC) at approximately 106 cells per dish. The assay was performed according to Levine *et al*. [45] using the classical derivatizing agent, 2,4-dinitrophenylhydrazine (DNPH). First, cells were collected and protein assay was conducted to normalize samples. Cells were then centrifuged at 200 x g for 5 min at 4 °C. The pellets were resuspended in monobasic sodium phosphate (100 mM) buffer (pH=6.8) containing 3 % sodium dodecyl sulfate. A 500 μL sample at 1 mg/mL was made and then 10 mM of DNPH in 100 μl of 2N HCl was added. The samples were then vortexed followed by a 1h-incubation at room temperature in the dark. The protein was precipitated with equal volume of trichloroacetic acid (20%, v/v) upon incubation on ice for 10 min followed by centrifugation for 5 min at 5,000 rpm. The pellets were washed with 1 mL of ethanol and ethyl acetate (1:1,v/v), sonicated, and centrifuged for 10 min at 5000 rpm, then they were resuspended in the denaturing buffer, dissolved overnight at room temperature, and the absorbance was read at 360 nm. Carbonyl contents were expressed as nmol carbonyls/mg protein.

### 3.9. Statistical analysis

Each dose/time group contained seven to 10 observations and each experiment was repeated four times. Data are presented as mean ± S.E.M. Except for the control experiments (no test compounds were used), all data were analyzed by two-way ANOVA (ethanol dose x compounds) at each dose of compounds (0.1 and 1 μM). When significance (P<0.05) was observed, one- way (test compound) ANOVA was conducted to determine whether test compound treatments alter dependent variables (cell viability, protein carbonyls, and MDA) at each dose of ethanol (100 and 200 mM) followed by a *post hoc* Tukey test to identify which compound treatments made the differences. For the control experiments, two-way ANOVA (ethanol dose x EW or ethanol condition) followed by a *post hoc* Tukey test was conducted to identify whether dependent variables differ between ethanol treatment and EW conditions.

## 4. Conclusions

The HT-22 cell line is a widely used paradigm for the study of neuroprotective agents, such as estrogens, against a variety of toxic insults, although different modes of action (genomic vs. nongenomic) have been reported for this cellular model [46,47]. We have previously shown that E2 treatment produced significant neuroprotection against EW in this *in vitro* model. The goal of the present exploratory studies was to examine the efficacy of non-steroidal and simple phenolic antioxidants, specifically BHT and OP, to prevent oxidative stress induced by EW. Computational chemistry [30–32] and a well-established cell-free assay of LPO [28,29] have also been done to assess the “pure” antioxidant potency of our test compounds. We concluded that BHT possessed the highest antioxidant potency (Table 1), and, thus, was expected to produce the maximal neuroprotection among the test compounds on the cell line.

We showed that EW produced more damage to the cells than ethanol exposure *per se* (Figure 2). When the cells were treated with E2, BHT or OP, a significant and dose-dependent reduction of cell death, LPO, and protein carbonylation was obtained after EW. However, contrary to theoretical prediction and cell-free measurements of antioxidant potencies, the three compounds were practically equipotent under the experimental conditions used in our *in vitro* studies (Figures 3–5). During these experiments, antioxidant treatment was done simultaneously with the 24-h ethanol insult. When the cells were exposed to the antioxidants during the 4-h EW only, the outcome was not significantly different (data not shown) from that obtained with the co-treatment schedule. Therefore, it may be sufficient to treat the cells with an antioxidant during EW only to alleviate the adverse effects of this neurotoxic insult. This finding will be explored in another study. On the other hand, an E2 derivate without free phenolic OH (3OBu-E2, Figure 1) had no effect on cell survival, MDA, and protein carbonyl formation, further implying the crucial role of free radical scavenging during EW to achieve neuroprotection. The lack of correlation between predictions derived from an *in silico* study and a cell-free assay in terms of antioxidant potency suggest, however, that it is highly unlikely that a single mechanism (i.e., the free-radical scavenging antioxidant action probed by our investigation) is responsible for the observed reduction of EW-initiated oxidative events with these phenolic compounds. Specifically, while E2 may be a less potent antioxidant than BHT, its protective effects may also involve a variety of genomic and non-genomic mechanisms implicated in estrogen neuroprotection [27]. Estrogenicity of OP has also been demonstrated [48] and it may contribute to the observed neuroprotection. It is worth emphasizing that the mechanism of neuroprotection observed in the HT-22 cell line has been proposed *via* both genomic and non-genomic routes [46,47]. In addition, these compounds may mediate neuroprotection by an interaction with the redox modulatory site at the NMDA receptor complex [49], although, there has been conflicting reports on the expression of functional NMDA in the HT-22 cell line [50,51].

In conclusion, our findings presented here imply that free-radical scavenging may not be the sole factor responsible for the observed neuroprotection during EW. Moreover, further studies are also needed to establish, whether the HT-22 line is indeed a suitable model for *in vitro* screening of antioxidants against EW-related neuronal damage.

## Figures and Tables

**Table 1.** Calculated BDE of the phenolic O–H and a cell-free LPO inhibitory potency of E2, OP and BHT, respectively.

Compound	BDE (kcal/mol)	IC_50_ (μM)	
		FTC	TBARS
**E2**	81.1	11.8 ± 1.6	3.9 ± 0.4
**OP**	80.7	130.0 ± 18	64.8 ± 5.3
**BHT**	77.0	4.4 ± 0.6	3.5 ± 0.5

BDE and IC_50_ values were determined as described in Section 3.
